# Transcriptome analyses shed light on floral organ morphogenesis and bract color formation in *Bougainvillea*

**DOI:** 10.1186/s12870-022-03478-z

**Published:** 2022-03-04

**Authors:** Wenping Zhang, Qun Zhou, Jishan Lin, Xinyi Ma, Fei Dong, Hansong Yan, Weimin Zhong, Yijing Lu, Yuan Yao, Xueting Shen, Lixian Huang, Wanqi Zhang, Ray Ming

**Affiliations:** 1 Center for Genomics and Biotechnology, Fujian Provincial Key Laboratory of Haixia Applied Plant Systems Biology, Fujian Agriculture and Forestry University, 350002 Fuzhou, Fujian China; 2 Xiamen Botanical Garden, 361000 Xiamen, Fujian China; 3 College of Life Sciences, Fujian Agriculture and Forestry University, 350002 Fuzhou, Fujian China; 4 College of Crop Sciences, Fujian Agriculture and Forestry University, 350002 Fuzhou, Fujian China; 5 Department of Plant Biology, University of Illinois at Urbana-Champaign, 61801 Urbana, IL USA

**Keywords:** Gene annotation, Floral organ identity genes, Betalain, Co-expression

## Abstract

**Background:**

*Bougainvillea* is a popular ornamental plant with brilliant color and long flowering periods. It is widely distributed in the tropics and subtropics. The primary ornamental part of the plant is its colorful and unusual bracts, rich in the stable pigment betalain. The developmental mechanism of the bracts is not clear, and the pathway of betalain biosynthesis is well characterized in *Bougainvillea*.

**Results:**

At the whole-genome level, we found 23,469 protein-coding genes by assembling the RNA-Seq and Iso-Seq data of floral and leaf tissues. Genome evolution analysis revealed that *Bougainvillea* is related to spinach; the two diverged approximately 52.7 million years ago (MYA). Transcriptome analysis of floral organs revealed that flower development of *Bougainvillea* was regulated by the ABCE flower development genes; A-class, B-class, and E-class genes exhibited high expression levels in bracts. Eight key genes of the betalain biosynthetic pathway were identified by homologous alignment, all of which were upregulated concurrently with bract development and betalain accumulation during the bract initiation stage of development. We found 47 genes specifically expressed in stamens, including seven highly expressed genes belonging to the pentose and glucuronate interconversion pathways. *BgSEP2b*, *BgSWEET11*, and *BgRD22* are hub genes and interacted with many transcription factors and genes in the carpel co-expression network.

**Conclusions:**

We assembled protein-coding genes of *Bougainvilea*, identified the floral development genes, and constructed the gene co-expression network of petal, stamens, and carpel. Our results provide fundamental information about the mechanism of flower development and pigment accumulation in *Bougainvillea*, and will facilitate breeding of cultivars with high ornamental value.

**Supplementary information:**

The online version contains supplementary material available at 10.1186/s12870-022-03478-z.

## Introduction

*Bougainvillea* originates in South America and belongs to the family Nyctaginaceae in the order Caryophyllales. The genus *Bougainvillea* Juss has 18 species, but only three species, *B. glabra* Choisy, *B. spectabilis* Wild and *B. peruviana* H. & B., have high ornamental values, from which *B. glabra* Choisy and *B. spectabilis* are the most showy ornamentals, that are widely distributed in the tropics and subtropics of the word [1, 2]. As an ornamental crop, *Bougainvillea* has not only bright and colorful flowers, but also has the effect of purifying air and noxious gas removal [3]. Meanwhile the extracts of roots and leaves of *Bougainvillea* contains antiviral proteins with antiviral characteristics, especially for tobacco mosaic virus (TMV) [4] and tomato spotted wilt tospovirus (TSWV) [5]. Plants in this genus also have been used in traditional medicine. Extracts of *Bougainvillea* has antinociceptive and anti-inflammatory effects [6], antihyperglycemic activity with the antidiabetic potential [7–9], and effective ingredients in prevention of neurological disorders [10–12].

The inflorescences of *Bougainvillea* are axillary, three-flowered umbel, and each floral unit consist of three abnormal leaves—bracts and three flowers, and each flower pedicel attached to the central midrib of the bract [2]. The abnormal floral organs may be regulated by the genes from floral ABCDE model, which proposes that floral organs identity are defined by five classes of genes (A, B, C, D, and E) from MADS-box and AP2/ERF gene family [13, 14]. Generally, the main ornamental organ is petals in *angiosperms*, which are controlled by A, B and E classes genes [15–17]. The B and C classes organ identity genes require the activities of E class genes, and they initiate in stamen development. C and E classes genes regulate carpel development [17]. The D class genes regulate ovule development [18, 19].

The organs with ornamental values of *Bougainvillea* are the floral bracts enriched with betalains, which are water-soluble and stored in vacuoles, showing brilliant color in flowers or fruits of species in the order Caryophyllalles [20]. They are usually used as natural colorant extracting from red beet because they are more stable than anthocyanins. The betalains are derive from tryrosine, and finally form betaxanthin and betacyanin after a series of enzymatic and spontaneous reaction [21].

The L-DOPA and betalamic acid are crucial substrate for betaxanthin and betacyanin. L-DOPA is catalyzed by tryrosine hydroxylation and tyrosinase or PPO form tryrosine in two ways [22–25]. Betalamic acid is catalyzed by DOPA 4,5-dioxygenase (DODA), which convert L-DOPA into 4,5-seco-dopa and spontaneous chemical reaction [22]. When L-DOPA is catalyzed by DOPA decarboxylase form dopamine, it enters the betaxanthin synthesis pathway. Dopamine directly binds with betalamic acid or it is catalyzed by catechol O-methyltransferase (COMT) after a spontaneous reaction and then forming miraxanthin-V and 3-methoxytyramain-betaxanthin, respectively. They are a form of betaxanthin [26, 27]. After L-DOPA is catalyzed by tryrosine oxidation or CYP76AD1 (R) produce Cyclo-Dopa, they are involved in glucosylation by the action of GTs [25, 28]. Betanidin 6-O-glucosyltransferase (B6GT) modified betanidin form Gomphrenin-I, and Betanidin 5-O-glucosyltransferase (B5GT) modified betanidin to betanin and form Lampranthin-II, while cyclo-DOPA 5-O-glucosyltransferase (cDOPA-5GT) converts Cyclo-Dopa into Cyclo-Dopa 5-O-glucoside and forms Lampranthin-II and Celosianin-II. These products are betacyanin [20, 29–32]. Besides biosynthesis pathway genes, some transcription factors are involved in regulation of betalain biosynthesis. In beet, BvMYB1 (Y) is involved in betalain pathway by regulating BvDODA1 and BvCYP76AD1 [33].

To date, no reference genome of *Bougainvillea* has been reported. Similarly, genetic studies on the mechanisms underlying floral organ morphology and development as well as betalain biosynthesis in *Bougainvillea* are scarce. In this study, we generated PacBio Iso-Seq and Illumina RNA-Seq data obtained from various organs and stages in petals, stamens, carpels, and bracts, and *de novo* assembled the expressed protein-coding genes to provide novel insights into key floral genes from the ABCE model participating in bract development; we also identified genes involved in betalain biosynthesis in *Bougainvillea*, and ascertained the gene co-expression network of petals, stamens, and carpels.

## Materials and methods

### Genome size analysis, plant RNA-Seq library construct and HiSeq 2500 sequencing

The urban greening commercial cultivar in southern China, *Bougainvillea glabra* “New River”, was maintained at FAFU greenhouse. It was used for genome size analysis, transcriptome analysis. Fresh young leaves of *Bougainvillea* “New River” were used for genome size analysis by flow cytometry. The genome size of each sample was estimated with four replications, and the details of the method and data analysis were described in the published article [34]. The use of plants in the study are comply with relevant institutional, national, and international guidelines and legislation.

The petals, stamens, carpels and five development stages (the length of bract with 0.5 cm, 1.0 cm, 2.0 cm, 2.5 cm, and 3.5 cm) of bracts with 2 replicates were sampled and isolated total RNA with total RNA extract kit (TianGen, DP430). The total RNA were quantified and the quality was assessed using an Agilent 2100 Bioanalyzer (Agilent, http://www.agilent.com). Then the RNA samples were used to construct RNA-seq libraries, and follow the protocol of kit (NEBNext Ultra RNA library Prep Kit). These libraries were sent to Beijing Novegene Bioinformatics Technology Co., Ltd for Illumina HiSeq 2500 high through sequence.

### Iso-Seq library preparation, PACBIO sequencing and analysis

The RNA of leaves, bracts and flowers were extracted separately, and then mixed equally according to total RNA amount of each sample for construct one SMRT cell library (0.5–10 kb). The samples were sent to Beijing Novegene Bioinformatics Technology Co., Ltd for library construct and Pacbio RSII sequence, and the library underwent SMRT sequencing using two SMRT cells. Subreads were filtered and subjected to CCS using the SMRT Analysis Server 2.2.0 (Pacific Biosciences of California, Inc). CD-hit (v4.6.8) [35] with identify 0.85 were used to filter redundancy isoforms.

### Coding genes annotation and differentially expression genes analysis

The RNA-seq data from HiSeq 2500 were filter by NGS QC Toolkit. Trinity-v2.8.4 [36] were used to assemble transcriptome data and analyze differentially expressed genes of 5 bract development stages. We assembled and annotated coding genes in *Bougainvillea* using RNA-Seq and Iso-Seq data from HiSeq 2500 and Pacbio RSII platform, respectively. We got 498,155 isoforms from RNA-Seq data (bract, petal, stamen, and carpel) by trinity assembly, and 257,704 isoforms were kept by CD-hit (v 4.6.8) with similarity thresholds of 0.85 to remove redundancy sequence. And there are 24,840 long reads from Iso-Seq (flowers and leaves tissues) after filter low quality sequence and corrected by short reads from RNA-Seq data. And 10,916 long reads were kept after CD-hit (i_0.85). Then the 257,704 isoforms from RNA-Seq data and 10,916 long reads form Iso-Seq platform were combine and redundancy sequence were removed. And 52,678 proteins were kept by TransDecoder predicated based on Pfam and Swissprot databases (https://github.com/TransDecoder/TransDecoder/wiki). Then the 55,988 proteins were further blastp by NR database and themselves in order to remove similar and negative proteins, and some filter jobs were made by perl scripts in linux.

The differentially expressed genes were analyzed by Trinity (v.2.8.6) and calculated by edgeR (*p* < 0.001) [37]. All heatmaps were drawn by R script (pheatmap(log2(Exp+1), scale="row”, cluster_cols=F, cellwidth=30, cellheight=15, fontsize=12, color = colorRampPalette (c(“white”,“red”)) (100))) or c(“green”,“white”,“red”), the parameters of cell width, cell height, font size were changed according to the number of genes and samples.

### Comparative genome analysis

The evolutionary history of *Bougainvillea* was not reported before, and proteins of *Bougainvillea* and other reported seven species were used to speculate the evolutionary time.Proteins and cds of seven species (*Spinacia oleracea*, *Beta vulgaris*, *Carica papaya*, *Arabidopsis thaliana*, *Vitis vinifera*, *Nelumbo nucifera*, *Oryza sativa*) were chosen to construct phylogenetic tree, and all data were download at ncbi (https://ftp.ncbi.nlm.nih.gov/genomes/refseq/plant/). The single-copy orthologous genes of all species were identified using OrthoMCL (v.2.0) [38]. The phylogenetic tree of seven species was construct by iqtree (v.1.6.3) [39] with single-copy orthologous genes, and ultrametric tree was drawn by the *Café* software (v.4.2) [40]. The *Arabidopsis thaliana* and *Carica papaya* divergence time (68–72 million years ago) [41] was applied as calibrators.

### Expression experiment

Real-time qPCR was designed to verify the expression trend of A, B, C, D, and E classes genes and then they were compared with transcriptome data. Mature leaves and five development stages of bracts, petals, stamens, and carpels were collected for Real-time qPCR. The primers were designed by Primer 5.0, and the primer sequences were listed in Table S1. Real-time qPCR (RT-qPCR) was performed on an Roche LightCycler 480 with SYBR Premix ExTaqII (Takara), and relative expression data were quantified by 2^−△△CT^ method [42] and normalized by 18s rRNA.

### Co-expression network analysis

The weighted gene correlation network analysis (WGCNA) (v 1.61) package in R [43, 44] was used to construct the gene co-expression network analysis for *Bougainvillea* based on the gene-level FPKM data from differentially expressed genes during the 5 bract developmental stages. Module detection was performed using the TOM-based similarity measure and the dynamic tree cutting algorithm to cut the hierarchal clustering tree and defined modules as branches from the tree cutting. The minimum number of genes per module was set as 30 genes by default, and the threshold of module merging correlation for eigengene similarity was 0.8. For the module-tissue association analysis, the eigengene value was calculated for each module and used to test the association with each tissue type. The total connectivity and intramodular connectivity, kME, and kME-p-value were calculated and represent the Pearson correlation between the expression level of that gene and the ME. Additionally, genes with highest degree of intramodular connectivity within a module are referred to as hub genes. The networks were visualized using Cytoscape_v.3.8.0 [45].

## Results

### *De novo* assembly of transcripts and annotation of coding genes

The mean genome size of cultivar species *Bougainvillea glabra* “New River” was about 3.04 Gb (*n* = 4) (Supplementary Figure S1). We present a transcriptomic analysis by combining PacBio Iso-Seq and Illumina sequencing technologies, aiming to (a) obtain accurate and full length transcripts; and (b) providing quantitative information for expression analysis. The coding genes number of RNA-Seq assembled and Iso-Seq data annotated were 257,704 isoforms and 24,840 long reads, respectively. The integrity of proteins from RNA-Seq assembled and Iso-Seq was 91.2 (S:65.2%; D:26.0%) and 40.6% (S:20.7%; D:19.9%), respectively. In addition, 23,469 proteins were retained after merging all proteins from the two platforms and removing redundancy (Table 1).

**Table 1:** Characteristics of respondents (*N* = 511)

Isoforms		C	S	D	F	M
NGS	257,704	91.2%	65.2%	26.0%	6.1%	2.7%
Iso-Seq	10,916	40.6%	20.7%	19.9%	2.5%	56.9%
NGS+ Iso-Seq	55,988	89.8%	85.5%	4.3%	7.3%	2.9%
Coding genes	23,469	89.2%	87.7%	1.5%	7.8%	3.0%

C: Complete BUSCOs; S: Complete and single-copy BUSCOs; D: Complete and duplicated BUSCOs; F: Fragmented BUSCOs; M: Missing BUSCOs

### Phylogenetics analysis of representative angiosperm species

**Fig. 1:**
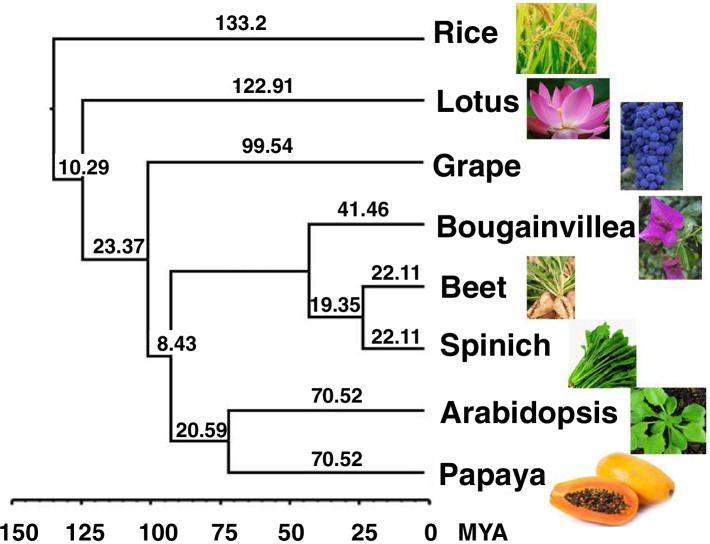
The species evolution analysis of *Bougainvillea.* The tree was constructed by neighbor-joint method. Species including spinach (*Spinacia oleracea*), beet (*Beta vulgaris*), papaya (*Carica papaya*), Arabidopsis (*Arabidopsis thaliana*), grape (*Vitis vinifera*), lotus (*Nelumbo nucifera*), rice (*Oryza sativa*)

The evolutionary history of *Bougainvillea* has not yet been reported. We therefore used proteins of *Bougainvillea* and seven other species to elucidate its evolutionary history. *Bougainvillea* belongs to the Nyctaginaceae family; however, no genome or protein library for this family has yet been reported. Therefore, the closest family protein libraries of spinach (*Spinacia oleracea*) from Chenopodiaceae and beet (*Beta vulgaris*) from Amaranthaceae were used to construct species trees. Four other species from four different families of the eudicot clade, including papaya (*Carica papaya*), *Arabidopsis* (*Arabidopsis thaliana*), grape (*Vitis vinifera*), lotus (*Nelumbo nucifera*). Monocot model species rice (*Oryza sativa*) was used as an outgroup species to construct the phylogenetic tree. The results showed that *Bougainvillea* was earlier than beet and spinach, and they diverged approximately 41.46 million years ago (MYA) **(**Fig. 1**)**.

### Expression of ABCE model genes during bract development

**Fig. 2:**
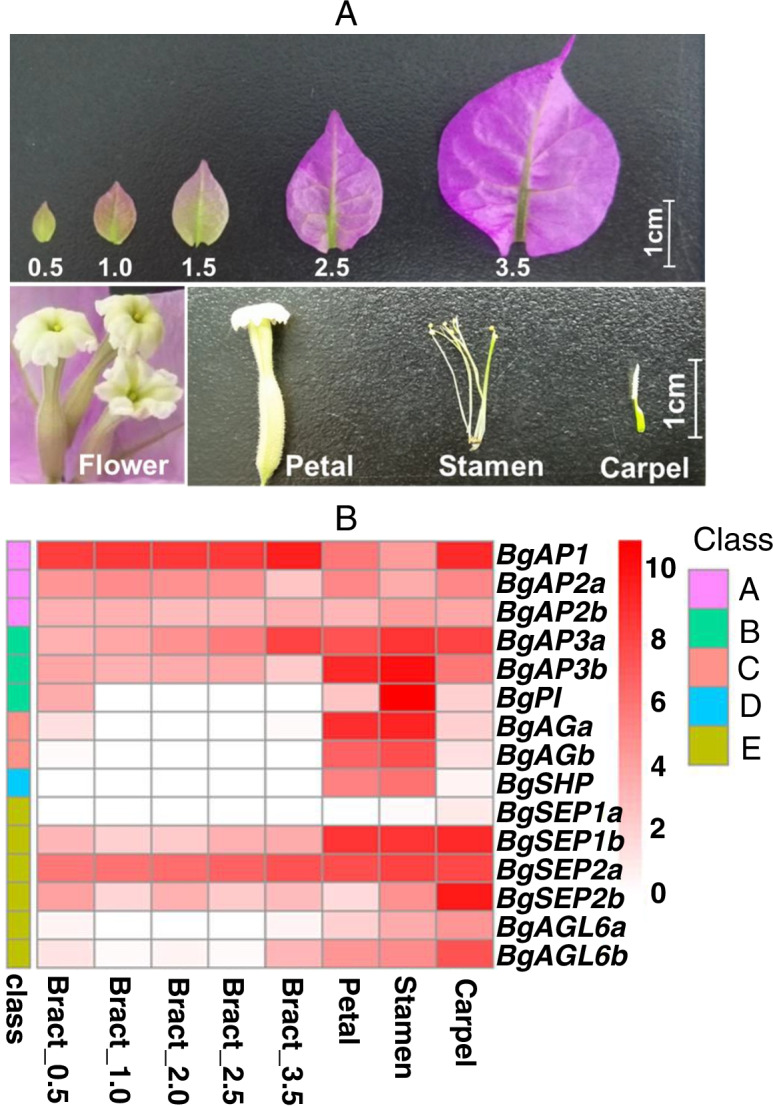
The expression analysis of flower organs in *Bougainvillea ***A**. The flower tissues samples, including 5 development stages of bract, petal, stamen, and carpel. **B**. The expression profile of ABCE model genes in floral organs (FPKM). **C**. The relative expression of ABCE model genes infloral tissues of *Bougainvillea*by RT-qPCR

The ABCE model genes play pivotal roles in floral organ development. We found 12 homologous genes of the ABCE flowering organ identity genes in *Bougainvillea*, as shown in Fig. 2. Among them, ten genes encoded MADS-box transcription factors; two genes were of *AP2* transcription factors (A-class). Expression profile analysis showed that *APETALA1* (*BgAP1*) in *Bougainvillea* showed high expression levels in bracts and petal tissues. The two homologous genes of *AP2* showed varying expression patterns. *BgAP2a* was highly expressed in bracts, petals, and carpels, whereas *BgAP2b* was also highly expressed in stamens. The B-class genes including *BgAP3* and *BgPI*. They showed the highest expression level in stamens by RNA-seq analysis, and *BgAP3* also showed high expression in petals and carpels by calculating the relative expression level (Supplementary Figure S2). The C-class gene *BgAG*, and D-class gene *BgSHP* showed higher expression levels in petals and stamens, and low or no expression in bract tissues. We also found they showed higher expression level in carpels by RT-qPCR (Supplementary Figure S2). There were four homologous genes from E-class, among which *BgSEP2a* and *BgSEP2b* showed high expression levels in bracts and petals tissues (Fig. 2B).

### Transcriptomes of bracts at different developmental stages

**Fig. 3:**
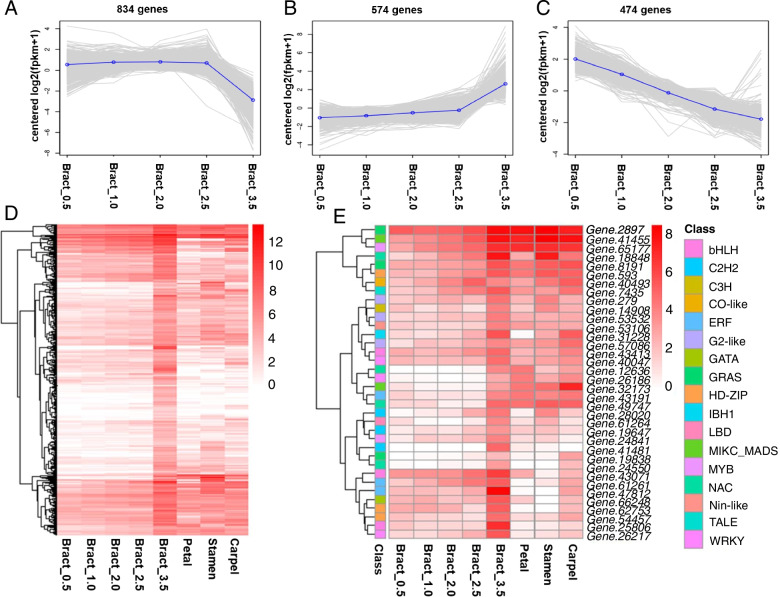
The expression trend and profile of 1882 differentially expression genes (DEGs) in bract. A-C. The three clusters of DEGs, and 574 genes in the up-regulated cluster. D. The expression profile of 574 up-regulated genes at 5 developmental stages of bracts, petal, stamen and carpel. E. The expression profile of 36 transcription factors of the 574 up-regulated genes. The data in the heatmap was normalized by log2(FPKM+1)

The bract is the main ornamental element of *Bougainvillea*, and the mechanisms underlying its development are not yet clear. We calculated the expression levels of 23,469 genes from the whole-genome analysis of *Bougainvillea*, and 1882 differentially expressed genes (DEGs) were found during the five developmental stages of bracts (*p* < 0.001). They formed three subclusters depending on their differential expression trends. A total of 574 genes were significantly upregulated during bract development (Fig. 3D), including 36 transcription factors from 17 families. Among them, 12 transcription factors showed significantly higher expression levels in adult bract—bract 3.5 compared to other flower tissues, including three bHLH, two C2H2, two ERF, two HD-ZIP, one MYB, one GATA, and one WRKY transcription factors (Fig. 3E).

Kyoto Encyclopaedia of Genes and Genomes (KEGG) enrichment analysis revealed that the upregulated genes were mainly involved in amino acid metabolism (tyrosine metabolism and valine, leucine, and isoleucine degradation), biosynthesis of other secondary metabolites (phenylpropanoid biosynthesis, tropane, piperidine and pyridine alkaloid biosynthesis, anthocyanin biosynthesis), carbohydrate metabolism (galactose metabolism) (10), energy metabolism (carbon fixation in photosynthetic organisms and nitrogen metabolism), and metabolism of terpenoids and polyketides (carotenoid biosynthesis, monoterpenoid biosynthesis, and zeatin biosynthesis) (*p* < 0.05) (Supplementary Figure S3). The genes in the phenylpropanoid biosynthesis (14) and galactose metabolism (10) pathways were more highly enriched than those in other pathways.

There were eight genes of the phenylpropanoid biosynthesis pathway, and six of them (*Gene.3747*, *BgPRX52-2*, *BgCAD4*, *Bg4CL1*, *Gene.5465*, and *CYP98A3*) showed significantly higher expression in bracts than in other flower tissues (Supplementary Figure S4). *BgCAD4* and *Bg4CL1* are crucial genes for lignin biosynthesis, and lignin is the main component of the secondary cell wall of vascular tissues [46, 47]. Two genes (*Gene.26858* and *BgBGAL12*) out of ten were involved in galactose metabolism. One (*Gene.38630*) out of two anthocyanin biosynthesis genes and one (*NCED4*) out of three carotenoid biosynthesis genes showed particularly high expression levels in bracts.

### Highlights of betalain biosynthesis-related genes in bract of *Bougainvillea*

**Fig. 4:**
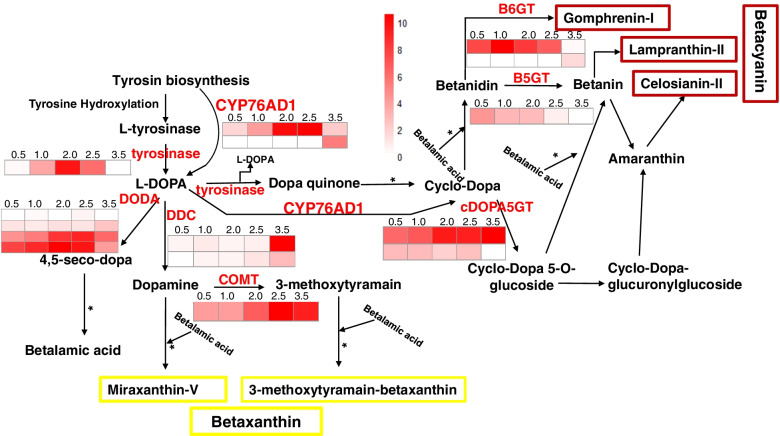
The expression analysis of betalain biosynthetic genes at 5 developmental stages of bract (from left to right was bract 0.5, bract 1.0, bract 2.0, bract 2.5, bract 3.5). The color from white to red indicated the expression level from low to high. The schematic diagram of betalain biosynthetic pathway was edited according to KEGG and reported reference[25]. “*” steads of spontaneous chemical reaction steps. The yellow borders represent the product of betaxanthin and the red borders represent the product of betacyanin. DODA: 4,5-DOPA-extradiol-dioxygenase; B6GT: Betalain 6-O-glucosyltransferase; B5GT: betanidin 5-O-glucosyltransferase; cDOPA5GT: Cyclo-DOPA 5-O-glucosyltransferase; COMT: Catechol O-methyltransferase; DDC: DOPA decarboxylase

We further identified 15 homologous genes encoding eight enzymes in the tyrosine-based biosynthetic pathway of betalain, including four genes encoding DODA, two *BgCYP76AD1* genes, two encoding DOPA decarboxylase, two encoding cDOPA5GT, two encoding B6GT, and two encoding COMT. Fourteen of these genes showed increased expression concurrent with bract development at the initial developmental stages (Fig. 4). In detail, *BgCYP76AD1* and the genes encoding B6GT, COMT, DDC, and DODA showed increased along with bract development and significantly high expression levels in the fourth (Bract 2.5) or fifth (Bract 3.5) developmental stages of bract; three genes (*DDC*, *B5GTb*, and *CYP76AD1a*) were found in the up-regulated cluster. The *TYR* gene encoding tyrosinase showed the highest expression peak at the third stage (Bract 2.0), while *B5GT* showed a declining trend during bract development. *cDOPA5GT* was expressed at all five developmental stages.

### Weighted gene co-expression network analysis (WGCNA) of different flower organs in *Bougainvillea*

**Fig. 5:**
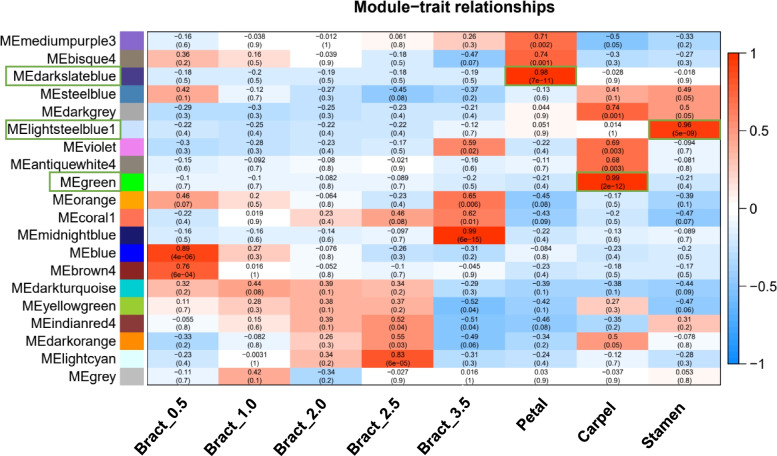
The weighted correlation network analysis (WGCNA) of floral tissues

A total of 12,987 genes (FPKM ≥ 3) were used to construct the co-expression network (Fig. 5). The MEdarkslateblue module (0.98) was chosen to analyse petal-specific co-expression genes; the MElightsteelblue1 module (0.96) was chosen to analyse stamen-specific co-expression genes; the MEgreen module (0.99) was chosen to analyse carpel-specific co-expression genes.

**Fig. 6:**
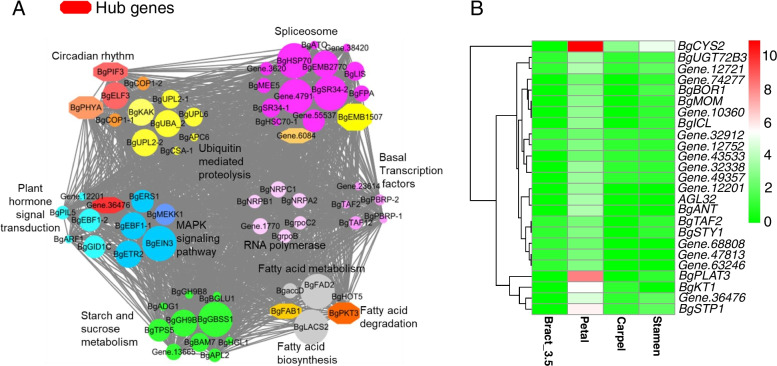
The co-expression network and express profile of petal high expression genes. **A**. Co-expression network of KEGG pathway genes (Pvalue <0.05). The size of circle indicated the expression level from high to low. The genes with octagon indicated hub genes, the color from red to yellow indicated the degree from high to low. **B**. The expression profile of 25 petal specific high expression genes.

The petal-specific module contains 1103 genes, 640 of which showed higher expression in petals than in other flower tissues. KEGG enrichment analysis indicated that 640 genes were mainly involved in the spliceosome (15), plant hormone signal transduction (11), ubiquitin-mediated proteolysis (9), and circadian rhythms (5) (Supplementary Figure S5). Three plant hormone genes were involved in the plant hormone signal transduction pathway, including auxin (AUX), ethylene (ETH), and gibberellin (GA). ETHYLENE INSENSITIVE 3 (*EIN3*) and two of its binding F-box proteins (EBF) were also found in this pathway. Seven genes showed significantly higher expression levels than other genes: *BgEIN3*, which is involved in plant hormone signal transduction and MAPK signalling, *Gene.47914*, *BgHSP70* and *BgSR34-2* of the spliceosome, *BgGBSS1* of starch and sucrose metabolism, and *BgLACS2* and *BgFAD2* of the fatty acid metabolism pathway. Furthermore, five genes related to the circadian clock were found in the module, including circadian clock gene *BgELF3*, photoperception genes *BgPHYA*, *BgPIF3*, *BgCOP1-1* and *BgCOP1-2* (homologous to *COP1* in *Arabidopsis*). *BgPHYA* and *BgELF3* are hub genes that interact with other genes (Fig. 6 A).

There were 25 genes that showed significantly high expression in petals (not expressed, or FPKM < 3, in other flower tissues), including seven transcription factors, two ERFs (*Gene.47813* and *BgANT)*, two ARFs (*Gene.12201* and *Gene.36476*), one Dof (*Gene.68808*), one TFII (*BgTAF2*), and one MADS-box (*BgAGL32*). *BgCYS2* and *BgPLAT3* showed significantly higher expression levels than the other genes in petals (Fig. 6B).

**Fig. 7:**
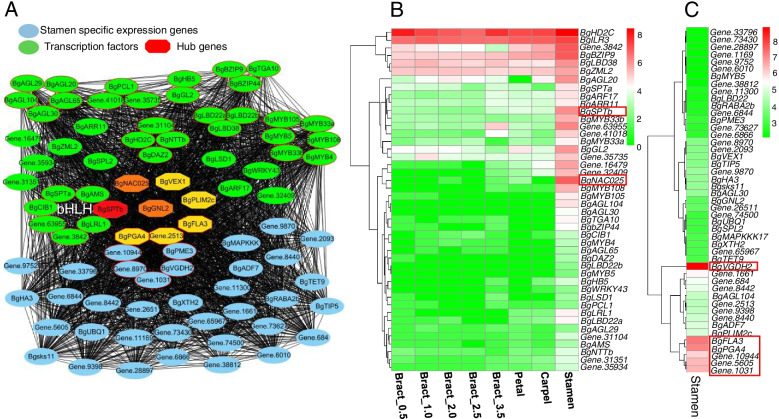
The co-expression network and expression profile of transcription factors and stamen specific expression genes. **A** The co-expression network of transcription factors and stamen specific expression genes. **B** The expression profile of transcription factors in the co-expression network. **C** The expression profile of 47 stamen specific expression genes

The stamen co-expression module contains 2,094 co-expression genes (113 TFs). A total of 1412 genes (75 TFs) exhibited higher expression levels in the stamen; upon analysis, they were found to mainly be enriched in genetic information processing and metabolism processes (*P* <0.05), including pentose and glucuronate interconversions (14), glycolysis/gluconeogenesis (16), fructose and mannose metabolism (9), proteasome (9), arginine and proline metabolism (8), cyanoamino acid metabolism (7), and lipoic acid metabolism (3) (Figure S6). There were 47 genes that showed specific expression in the stamen, 15 genes involved in KEGG pathways, and seven genes in the pentose and glucuronate interconversions pathway, including four genes (*Gene.1030*, *Gene.8930*, *BgPME3*, and *BgVGDH2*) encoding pectinesterase, two genes (*Gene.2513* and *Gene.10944*) encoding pectate lyase, and one gene (*BgPGA4*) encoding exopolygalacturonase. Co-expression network analysis (weight > 0.3) identified 44 transcription factors that interact with these specific expression genes (Fig. 7 A). The eight hub genes, including two transcription factors (*BgSPTb* and *BgNAC025*), also showed high expression levels in the stamen (Fig. 7B); the other six hub genes were stamen-specific genes. Expression analysis found that *BgVGDH2*, *BgPGA4*, *Gene.10944*, and *Gene.1031* of the pentose and glucuronate interconversion pathways, as well as *BgFLA3* and *Gene.5605* exhibited higher expression levels among these specific expression genes in the stamen (Fig. 7 C).

**Fig. 8:**
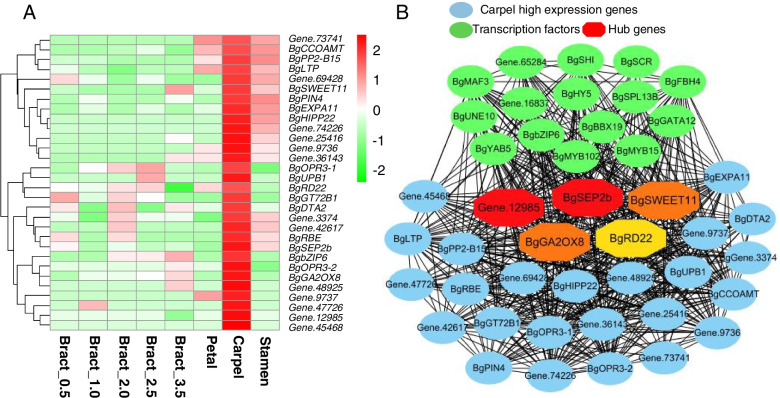
The expression profile of 30 carpel specific high expression genes and co-expression network. **A **The expression profile of 30 carpel specific high expression genes. The heatmap was drawn by R with log2(FPKM+1). **B** The co-expression network of transcriptions factors and carpel specific genes. The top 5 nodes (about 1%) were set to find hub genes. The color of hub genes from red to yellow indicated the degree from high to low

The carpel-specific module contains 380 genes, including 30 transcription factors, and 272 genes with higher expression levels in petals than in other flower tissues. These genes are involved in the MAPK signalling pathway (6), biosynthesis of secondary metabolites (17), and plant-pathogen interactions (5) (Figure S7). Thirty out of 272 genes showed significantly higher expressions in the carpel, and five genes (*BgRD22*, *BgCCOAMT*, *BgSEP2C*, *BgSWEET11*, and *BgPP2-B15*) showed the highest expression level in carpels as compared to other genes (more than four-fold) (Fig. 8 A). Two genes (*Gene.9736* and *Gene.9737*) encoding pectate lyase showed no expression in bract and significantly higher expression levels in carpel (4–12-fold higher than in the petal or stamen). The GA biosynthesis-related gene *GA2OX8* exhibited an exceedingly high expression level in the carpel (11–88-fold that of other flower tissues). The co-expression network (weight > 0.2) identified 16 transcription factors that interacted with these 30 carpel-specific high expression genes. *BgSEP2b*, *Gene.12985*, *BgSWEET11*, *BgGA2OX8*, and *BgRD22* were the top five hub genes in the network (Fig. 8 B).

## Discussion

In flowering plants, the development of the main ornamental parts is regulated by genes belonging to the classes A, B, and E [16, 17, 34]. The results showed that the expression profiles of floral organ-regulated genes of ABCE slightly differed from that of typical flowering plants. Bract formation related to A, B, and E class genes; A-class genes such as *BgAP2* showed lower expression levels in petals, indicating the possible formation mechanism of large colourful bracts and small dull petals. *STK* and *SHP* as D class genes specific to regulate ovule identification [17]. In *Bougainvillea*, we couldn’t identify any *STK* orthologous gene. The *STK* orthologous gene may be lost in *Bougainvillea*, or simply not annotated; this requires further genome sequencing for verification.

The *Bougainvillea* plant shows self-incompatibility. Previous reports have shown that B, C, and E-class genes regulate stamen development; C and E proteins complexes identify carpels; and D-class proteins specify ovule identity by interacting with E class proteins [15, 18]. In *Bougainvillea*, we calculated the expression level of A, B, C, D, and E-class genes by RNA-seq data and real time-qPCR. The expression analysis showed that B-class (*BgAP3a*, *BgAP3b*, and *BgPI*), C-class (*BgAG*), and D-class (*BgSHP*) genes showed high expression levels in stamens and carpel, while three out of four (*BgSEP1*, *BgSEP2b*, *BgAGL6*) E-class genes exhibited their highest expression levels in carpels, followed by stamen tissues (Fig. 3). From the expression profile, we found that C and D class genes showed lower expression levels in the carpel, while we proved they showed high expression in carpel by real time-qPCR (Fig. 3 C). E-class genes showed the highest expression in carpel tissues, which was consistent with real time-qPCR, indicating that the D-class genes may be functionally redundant in ovule identification, and E-class genes may play crucial roles in carpel identification. Due to the different calculate methods, the expression trend of some genes were inconsistent between RNA-seq data and real time-qPCR, but the overall gene expression trend of ABCE model genes were consistent as flowering plant.

Betalain is the main pigment in *Bougainvillea* flowers, which is more stable than anthocyanins in different PH [20]. However, the enzymes involved in the biosynthetic pathway are not as well understood as those of other plant pigments. Only eight homologous enzyme proteins were found in *Bougainvillea*. They showed different expression modes during the five bract development stages, which may be related to their different locations in the biosynthetic pathway of betalain. Transcription factors, such as MYB–bHLH–WD repeat (MBW) [48], participate in pigment biosynthesis in plant kingdom by regulating key genes in biosynthetic pathways. In betalain biosynthesis, MYB and WRKY transcription factors are also involved. For example, *BvMYB* regulates *CYP76AD1*/*R* and *BvDODA1* in beet [33], and *HmoWRKY40* regulates *HmoCYP76AD1* in Pitaya [49].

In petal-specific module, we found plant hormone signal transduction and circadian rhythms genes’ specific expression in petals. Two ERFs (*Gene.47813* and *BgANT*) and two ARFs (*Gene.12201* and *Gene.36476*) showed specifically high transcriptional level in petal, indicating the crucial role of ethylene and auxin in regulating petal development in *Bougainvillea*. Photoperception genes *BgCOP1-1* and *BgCOP1-2* were homologous with E3 ubiquitin-ligase COP1 regulating flowering in *Arabidopsis* [50], and they were found in petal-specific module, indicating their importance in petal development in *Bougainvillea*.

Stamen-specific model contains 47 specifically expressed genes, and seven of them encoding pectinesterase, pectate lyase, and exopolygalacturonase in *Bougainvillea*. The genes encoding pectinesterase are specifically expressed in stamens in wheat, and are involved in the regulation of male fertility [51]. The genes encoding exopolygalacturonase in maize are expressed in tissues associated with pollen development [52]. Pectate lyase degrades pectin in plants and contributes to the mechanical strength and physical properties of the primary cell walls [53]; some genes of this family are specifically expressed in pollen, such as *Late Anther Tomato 56* and *Late Anther Tomato 59*, expressed at maximal levels in mature anthers [54].

Previous research had demonstrated that *SEP2* was organ-identity genes and required for development of petals, stamens, and carpels in *Arabidopsis* [17]. In carpel co-expression network analysis, *BgSEP2b* showing high expression level in carpels and it was a hub gene interacted with many transcription factors and carpel-associated genes, suggesting it was a crucial gene in carpel development in *Bougainvillea*.

## Conclusions

We obtained 23,469 coding genes using RNA-seq and Iso-Seq data assembly. The genome evolution analysis showed that *Bougainvillea* was most closely related to spinach of the plant species analysed, and the two diverged approximately 52.7 MYA. Genes in ABCE model of flower development and betalain biosynthetic pathway genes were identified by homologous comparison. Bracts, the main ornamental organs of flowers, are regulated by A, B, and E-class genes. Betalain accumulation along with flower development, and the genes in betalain biosynthetic pathwayshowed an upregulated trend concurrent with bract development. Transcription factors, phenylpropanoids, and pigment biosynthesis pathway genes are involved in bract development. The genes of the spliceosome, plant hormone signal transduction, ubiquitin-mediated proteolysis, and photoperception and circadian rhythm pathways were co-expressed in petals. We found 47 stamen-specific genes in the stamen co-expression module; the genes encoding pectinesterase, pectate lyase, and exopolygalacturonase play crucial roles in stamen development. The stress response genes *BgRD22*, lignin biosynthetic gene *BgCCOAMT*, floral gene *BgSEP2b*, sugar transporter *BgSWEET11*, and *BgPP2-B15* showed higher levels of expression in carpels than did other genes. Finally, we found that *BgSEP2b*, *BgSWEET11*, and *BgRD22* as hub genes interacted with many transcription factors and genes in the carpel co-expression network.

## Supplementary Information

**Additional file 1: TableS1.** The real time-qPCR primers sequence of ABCE model genes. **Figure S1. **The genomic sizeof *Bougainvillea glabra* “New river”. The genomic size was about3.035Gb by the average of 4 replicates measure using flow cytometer. **FigureS2. **Therelative expression of ABCE model genes infloral tissues of *Bougainvillea*by real time-qPCR. **Figure S3. **KEGGenrichment analysis of 574 up-regulated genes at fivedevelopmental stages of bract. These genes mainly rich in metabolicpathway, biosynthesis of secondary metabolites, phenylpropanoid biosynthesis,etc. **Figure S4. **The expressionprofile of phenylpropanoid biosynthesis, galactose metabolism, anthocyaninbiosynthesis and carotenoid biosynthesis genes. The genes with red border indicatedthey had high expression level in that pathway. **Figure S5. **The top 20 ofKEGG enrichment pathway of 640 petal high expression genes. **Figure S6. **The top 20 ofKEGG enrichment pathway of 1412 stamen high expression genes. **Figure S7. **The top 20 ofKEGG enrichment pathways of 272 carpel high expression genes.

## Data Availability

All RNA-seq data of *Bougainvilean* reported in this paper have been deposited in the Genome Sequence Archive [55] in National Genomics Data Center [56], China National Center for Bioinformation / Beijing Institute of Genomics, Chinese Academy of Sciences, under accession number CRA004850 that are publicly accessible at https://ngdc.cncb.ac.cn/gsa.
