# Supplementary material for: Transcriptome analyses shed light on floral organ morphogenesis and bract color formation in Bougainvillea
Source: BMC Plant Biol. 2022 Mar 4;22:97. doi: 10.1186/s12870-022-03478-z (PMC8895829; doi:10.1186/s12870-022-03478-z)
Supplement: Supplementary file 1 — Additional file 1: TableS1. The real time-qPCR primers sequence of ABCE model genes. Figure S1. The genomic sizeof Bougainvillea glabra “New river”. The genomic size was about3.035Gb by the average of 4 replicates measure using flow cytometer. FigureS2. Therelative expression of ABCE model genes infloral tissues of Bougainvilleaby real time-qPCR. Figure S3. KEGGenrichment analysis of 574 up-regulated genes at fivedevelopmental stages of bract. These genes mainly rich in metabolicpathway, biosynthesis of secondary metabolites, phenylpropanoid biosynthesis,etc. Figure S4. The expressionprofile of phenylpropanoid biosynthesis, galactose metabolism, anthocyaninbiosynthesis and carotenoid biosynthesis genes. The genes with red border indicatedthey had high expression level in that pathway. Figure S5. The top 20 ofKEGG enrichment pathway of 640 petal high expression genes. Figure S6. The top 20 ofKEGG enrichment pathway of 1412 stamen high expression genes. Figure S7. The top 20 ofKEGG enrichment pathways of 272 carpel high expression genes. [file 12870_2022_3478_MOESM1_ESM.docx]

**Transcriptome analyses shed light on flower organs morphogenesis and bract color formation in *Bougainvillea***

Wenping Zhang ^1†^, Qun Zhou ^2^^†^, Jishan Lin ^1^, Xinyi Ma ^1,3^, Fei Dong ^1,3^, Hansong Yan ^1^, Weimin Zhong ^1^, Yijing Lu ^1,4^, Yuan Yao ^1,3^, Xueting Shen ^1^, Lixian Huang ^1^, Wanqi Zhang ^2^*, Ray Ming^5^*

^1^Center for Genomics and Biotechnology, Fujian Provincial Key Laboratory of Haixia Applied Plant Systems Biology, Fujian Agriculture and Forestry University, Fuzhou, Fujian, 350002, China.

^2^Xiamen Botanical Garden, Xiamen, Fujian 361000, China.

^3^College of Life Sciences, Fujian Agriculture and Forestry University, Fuzhou, Fujian 350002, China

^4^College of Crop Sciences, Fujian Agriculture and Forestry University, Fuzhou, Fujian, 350002, China.

^5^Department of Plant Biology, University of Illinois at Urbana-Champaign, Urbana, IL 61801, USA.

^†^ These authors contributed equally to this work

^*^Correspondence, Email: [rayming@illinois.edu](mailto:rayming@illinois.edu)

| Table S1. The real time-qPCR primers sequence of ABCE model genes | | |
| --- | --- | --- |
| Gene | Forward primers | Reverse primers |
| *BgAP1* | 5' GATCCAGAATTTAGAGCAACAA 3' | 5' ACAACCCAAGTGACAAGAGTAG 3' |
| *BgAP2a* | 5' AGGAGGCATATAATGAGGCAGA 3' | 5' CCAGTAATGGGAATGGGGAGAG 3' |
| *BgAP2b* | 5' TCTTCCCCCCCAAAAAAAAAAAA 3' | 5' GATGACTGGCAGAACTTGACCCC 3' |
| *BgAP3a* | 5' TTTCTCTCTTGTCTCGTACCCTC 3' | 5' TATTTACCTCGTTCAACTTCTGC 3' |
| *BgAP3b* | 5' GTTTCTCTTCTTATGGTCTCCG 3' | 5' GTTCTTTACCTTCTTTTTGCTA 3' |
| *BgAG* | 5' TGCTCAACCCTTCTCCCATCAT 3' | 5' AACAAACCCCCAAACACACTTC 3' |
| *BgPI* | 5' AGAAAGCTAAGGAAATCACTGT 3' | 5' GATCTCCATCTGCCTATCACGG 3' |
| *BgSHP* | 5' TTATGAATTATCTGTTCTTTGCG 3' | 5' CTGTTGTTTTCTGTTATGGTGCG 3' |
| *BgSEP1* | 5' GAGCAGAGCAGCTACAGGGAG 3' | 5' AAACCAGGGACTTGAGCATGAGT 3' |
| *BgSEP2a* | 5' CTTATGAACTCTCTATTCTCTGC 3' | 5' TGTCTTAATTGACCTAATTTGCC 3' |
| *BgSEP2b* | 5' ACAGGCAGGTGACGTTTGCTAAG 3' | 5' ACTCGTGCTGGAAAATGGGGGTT 3' |
| *BgAGL6* | 5' CCCTTATCGTCTTCTCTTCTCGC 3' | 5' GTGCCTCCATTTGCTCACTCATC 3' |
| *18S* | 5' CAGAACATCTAAGGGCATCACA 3' | 5' TAGTTGGTGGAGCGATTTGTCT 3' |


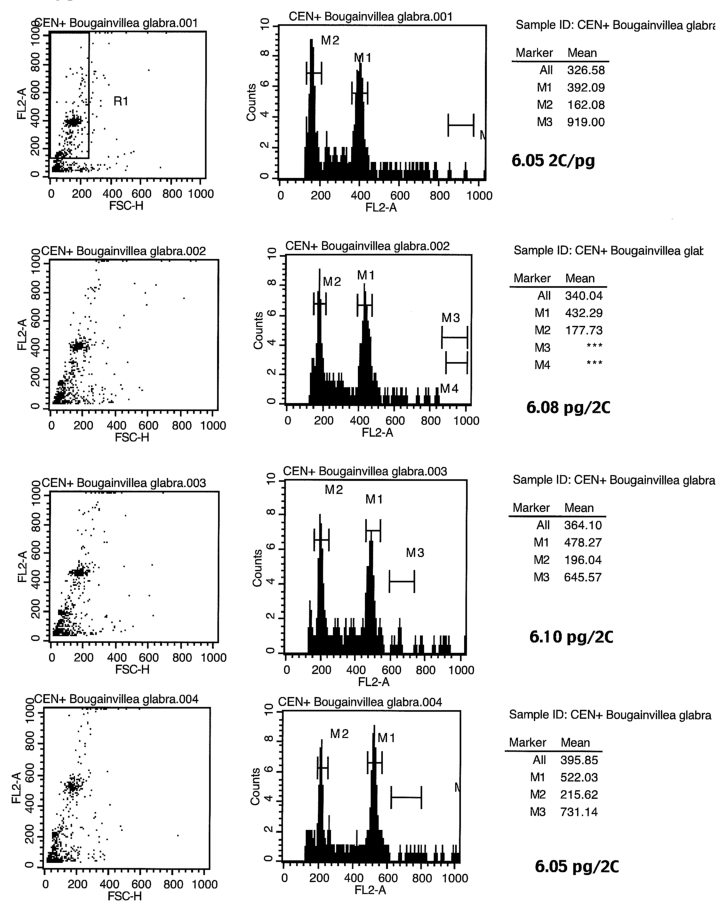


**Figure S1. The genomic size of *Bougainvillea glabra* “New river”.** The genomic size was about 3.035Gb by the average of 4 replicates measure using flow cytometer


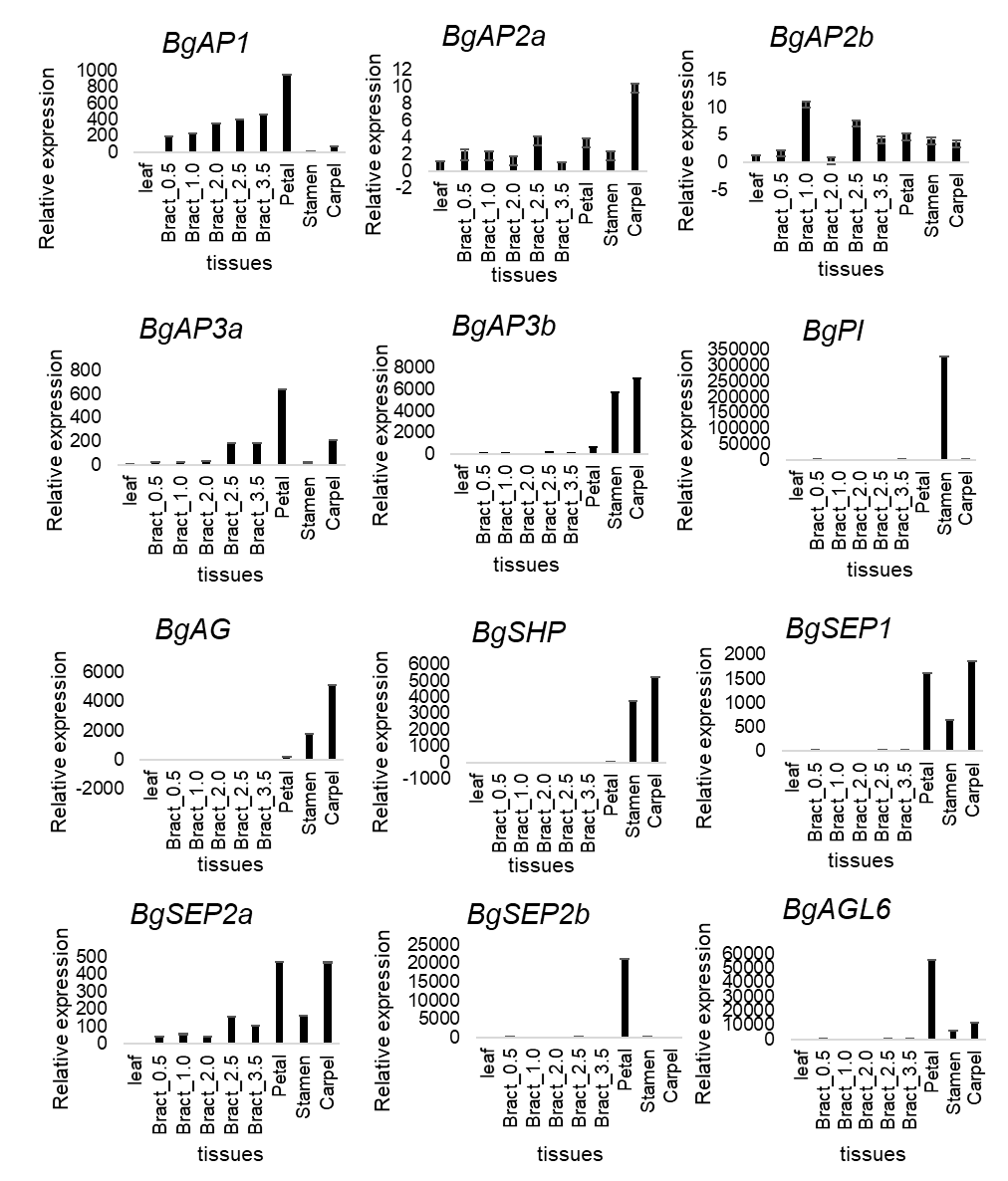


**Figure S2.** The relative expression of ABCE model genes in floral tissues of *Bougainvillea* by real time-qPCR.


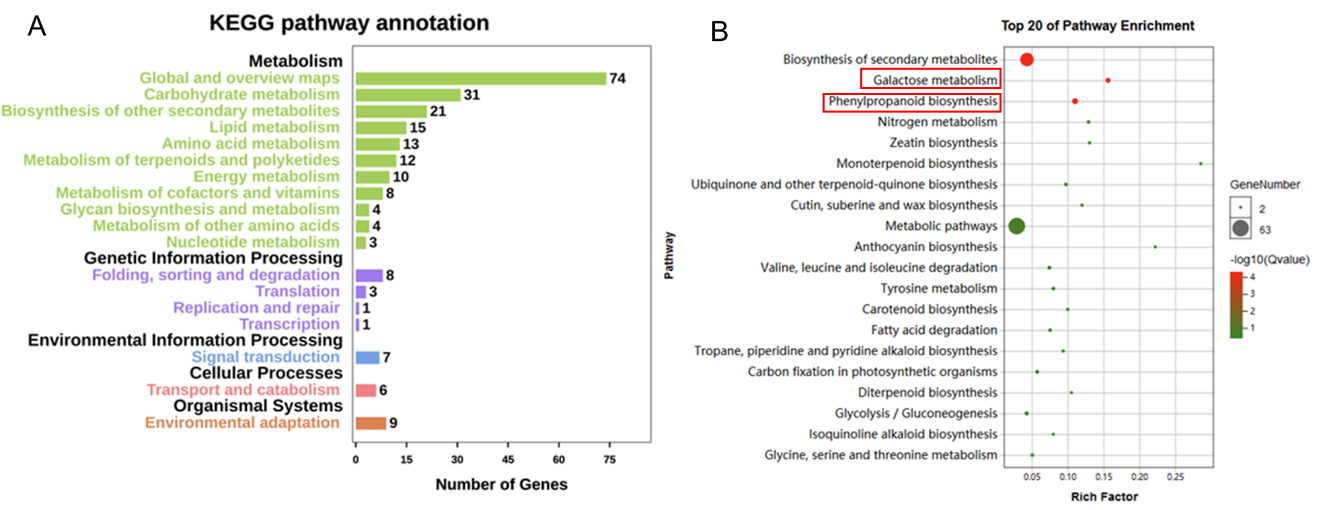


**Figure S3. KEGG enrichment analysis of 574** **up-regulated genes at five developmental stages of bract.** These genes mainly rich in metabolic pathway, biosynthesis of secondary metabolites, phenylpropanoid biosynthesis, etc.


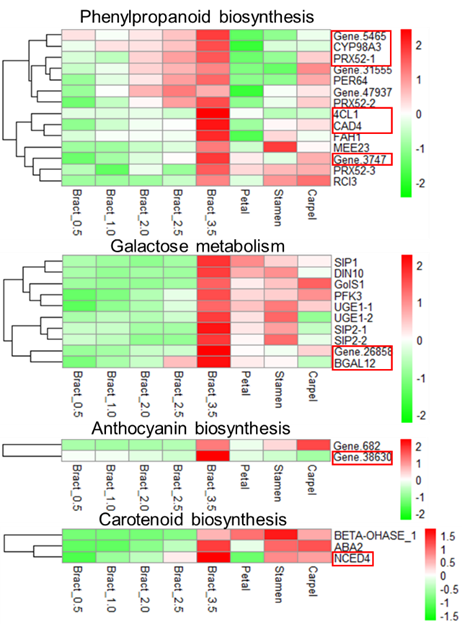


**Figure S4. The expression profile of phenylpropanoid biosynthesis, galactose metabolism, anthocyanin biosynthesis and carotenoid biosynthesis genes.** The genes with red border indicated they had high expression level in that pathway.


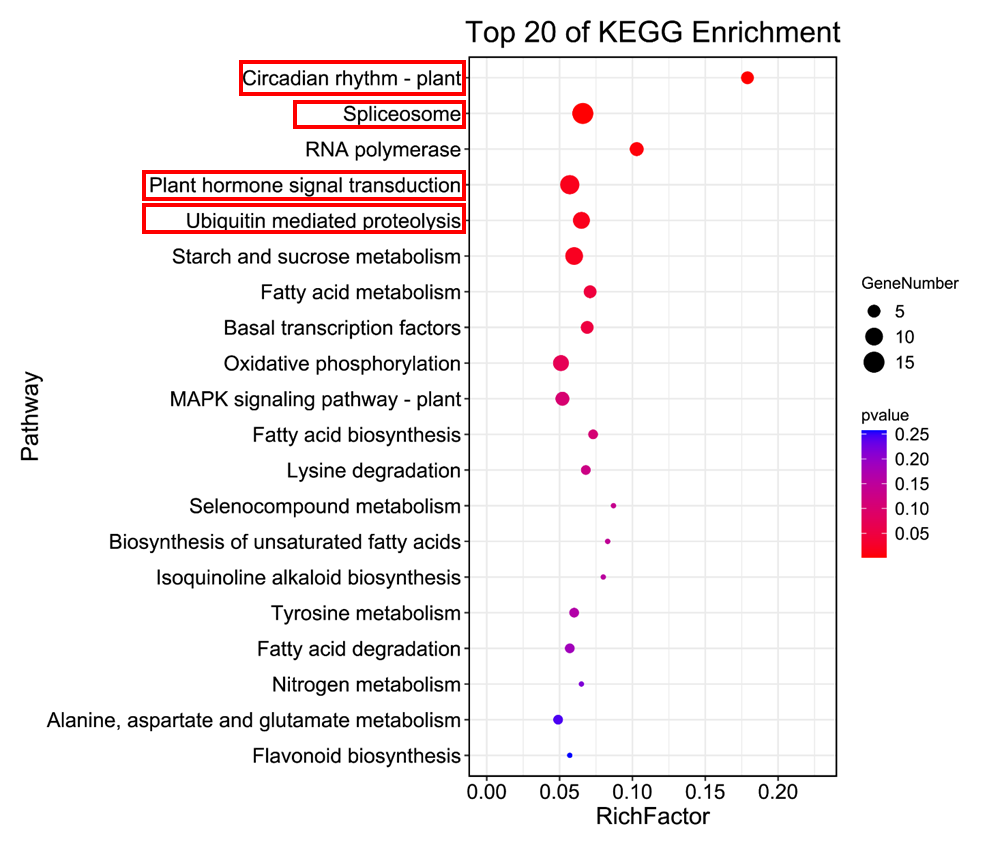


**Figure S5. The top 20 of KEGG enrichment pathway of 640 petal high expression genes.**


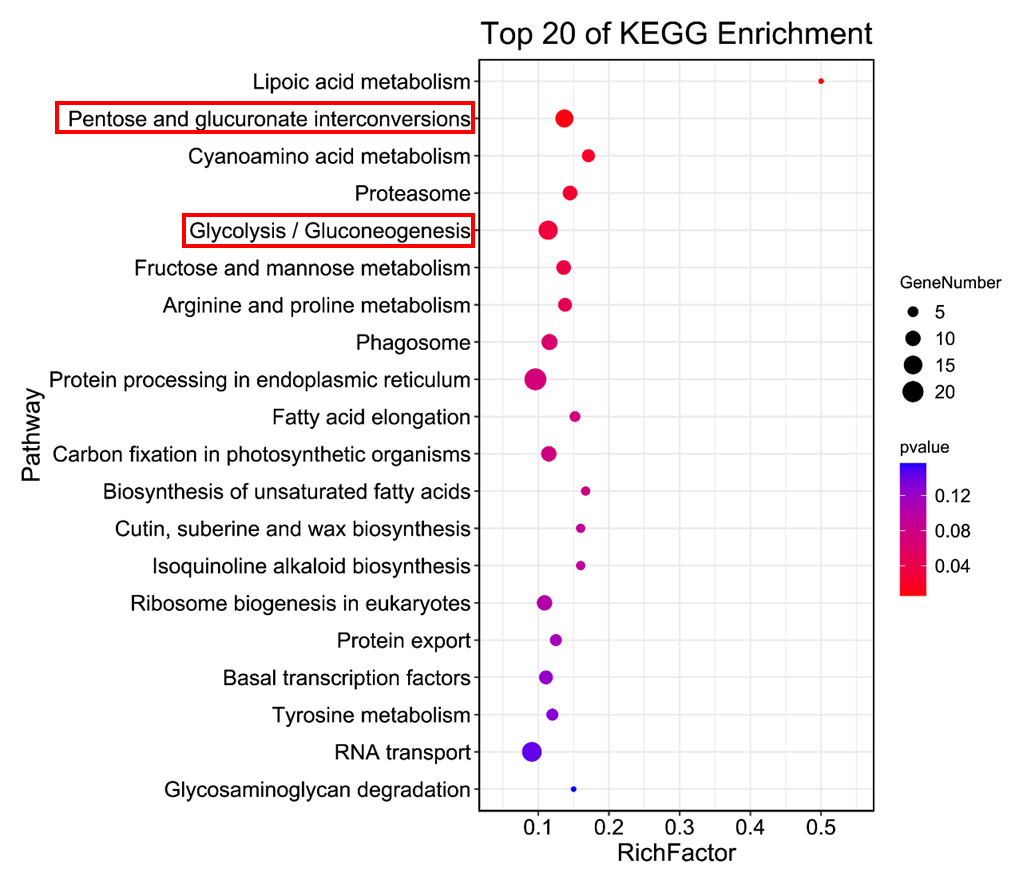


**Figure S6. The top 20 of KEGG enrichment pathway of 1412 stamen high expression genes.**


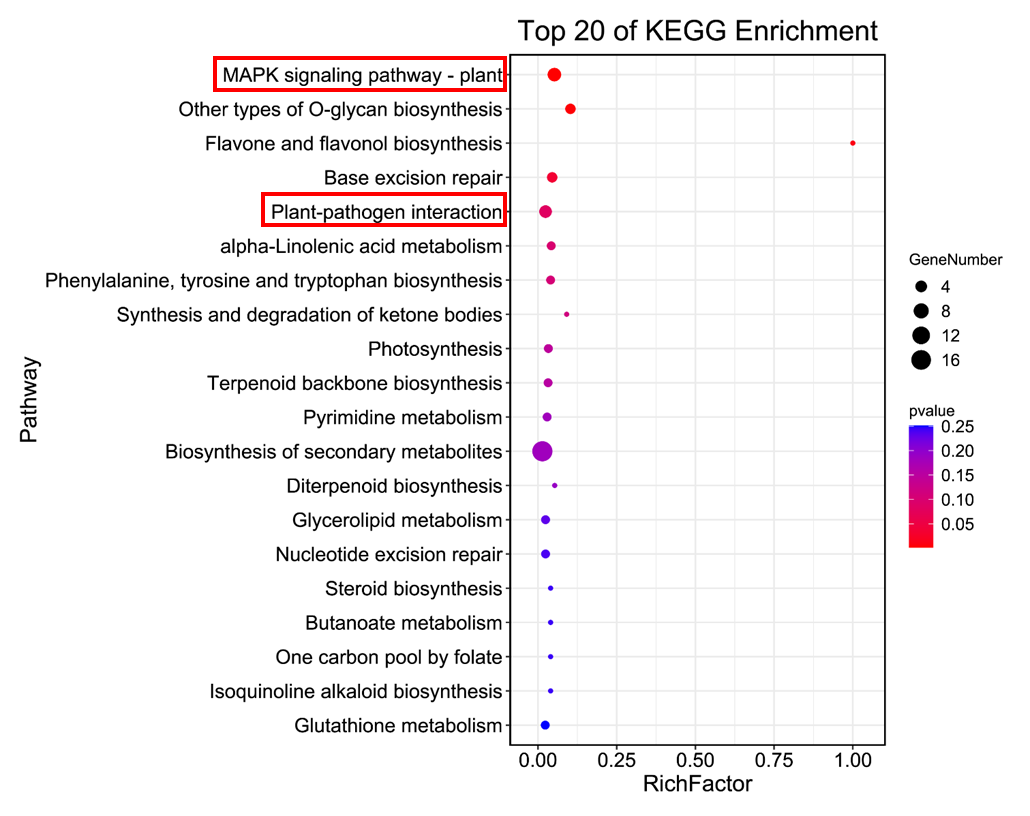


**Figure S7. The top 20 of KEGG enrichment pathways of 272 carpel high expression genes.**
